# 

**Published:** 1888-11-17

**Authors:** 


**Notice to Advertisers and Subscribers.**

All Advertisements, Orders for Copies of The Hospital, and Business Communications, should be addressed to The Publisher, *not to the Editor*, The Hospital, 38, Old Jewry, E.C.

A Scale of Charges will be found in the Advertisement Sheets, page iii.

**To Residents, Secretaries, and Matrons.**

The Editor will be glad to have the Regulations and each Report as issued by Asylums, Hospitals, Convalescent and Nursing Institutions, as well as those of other Charities, and an early copy in duplicate of all Appeals and Festival Notices, etc., addressed to *The Editor, The Lodge, Porchester Square, London, W.* Any superintendent, secretary, matron, or other chief official who regularly sends such information may be registered, on application to the Publisher, to receive free of cost a cover for binding The Hospital in half-yearly volumes.

### Scraps and Gleanings.

A concert was given at Hove Town Hall on October 22nd, in aid of the Sussex County Hospital.

The triennial festival dinner in aid of the funds of Charing Cross Hospital will take place on November 21st.; the Earl of Derby in the chair.

Charlotte Ellaby, M.D., is delivering a course of lectures on "Physiology" at Queen's College, Harley Street.

Chief Rabbi Gaster, well known as an author, preached the hospital sermon in the Birmingham Synagogue on October 28th.

John Grant was charged at Westminster Police Court last week with breaking out of the Aldershot Military Hospital. Prisoner turned the hospital uniform inside out, so that he appeared to be dressed in white flannel.

Stanley Hospital, Liverpool, is in debt to the extent of £4,000. Mr. Barkeley Smith has generously given £100 towards the liquidation of this debt on condition that nine other persons contribute a like sum before Christmas.

Brackley Cottage Hospital has lost a friend in the removal of Dr. Scott from that town. Not only did Dr. Scott give his services, but he provided drugs, &c.

On November 2nd, Alderman Cudlipp laid the stone of a new wing for Milton Infectious Hospital. It is to cost £900.

The children of the various schools at Northampton are giving the cots for the new children's ward at the infirmary. Each cot costs five pounds.

Windsor and Eton Royal Infirmary has received a legacy of £100 from the late Miss S. H. Hibberd.

The Rev. R. G. MacClelland has been appointed Chaplain of Suffolk General Hospital, in place of the Rev. C. Crossley resigned.

A concert was given to the patients of the Royal National Hospital, Ventnor, last week, by several of the ladies and gentlemen of the town.

The Mayor of Lancaster has visited and approved the site in Springfield Park proposed for the new infirmary for Lancaster. The site is now secured.

It is proposed to admit unmarried women to the Brighton and Hove Lying-in Institution. There is great opposition to the scheme.

The last meeting of the old committee of the Bournemouth Dispensary and Cottage has been held. A new committee has been nominated which is to take over the existing hospital on Jan. 1st, and then start the Victoria Hospital.

The cry of sectarianism has been got up against the Ulster Hospital, Belfast. It is utterly unfounded.

On Tuesday, November 20th, a private subscription dance will be given in aid of the Extension Fund of St. Mary's Hospital.

On December 1st, at the Holborn Restaurant, the annual dinner of the Dental Hospital of London will be held, Mr. J. S. Turner in the chair.

The Lumleian Lectures next year will be by Dr. John Harley, on "Enteric Fever."

Birmingham General Hospital has received £2,500 as the result of the late festival.

Dr. Zavadil, of Olmütz, is charged with poisoning his wife with arsenic.

Petersham has several cases of scarlet fever. It is proposed to open a temporary infectious hospital.

Sutton, Surrey, has started an ambulance for removing infectious cases to London, until it shall have secured a hospital of its own.

Dr. Nichols has resigned his post of medical officer at Croydon.

A concert was given at the Marlborough Rooms last week, in aid of the East London Hospital for Children.

The Lincoln Hospital Saturday Collection amounted to £332.

The Liverpool Hospital Sunday Fund amounted to £9,553, against £9,296 collected last year.

The Cheltenham Hospital Sunday Fund amounted to £537, against £645 collected last year.

The Leicester Hospital Sunday Fund has reached a thousand pounds.

The Birmingham hospital Sunday Fund has reached four thousand pounds.

Hospital Sunday at Newbury has resulted in £25.

The erection of a small-pox hospital for Londonderry, cost £700, has been approved.

Sir Rutherford Alcock has appealed in the *Times* for £2,000, to open the closed wards of the Royal Westminster Ophthalmic Hospital.

On December 31st Mr. W. C. Hatch's Amateur Dramatic Company will give a performance at Richmond in aid of the hospital.

Stratford-on-Avon is going to leave the boxes supplied by the Hospital Sunday Fund permanently in different shops.

Lady Sutton and the Hon. Miss Bridgeman held the collecting plates after the service in honour of the anniversary of Salop Infirmary.

Pembroke Hospital Sunday collections have amounted to about £230.

Dr. Black, late of Ballymena, has left £200 to the Cottage Hospital of that town.

Dr. Wheatley has been appointed Senior House Surgeon of Blackburn Infirmary, in place of Dr. Gifford, resigned.

The matron of the Bristol Hospital for Sick Children and Women has already issued an appeal for Christmas gifts.

It is proposed to register the Hospital Saturday Fund under "The Companies' Act," to give confidence to subscribers.

On Saturday last Lady Maryon Wilson laid the memorial of the Blackheath and Charlton Cottage Hospital.

It is proposed to move Birkby Fever Hospital to a more isolated site.

Lady William Lennox has written an article in the *Queen*, describing the Royal Hospital, Dublin.

The floor of the church of St. Andrew-by-the-Wardrobe has been discovered to be immediately over the graves of a number of persons supposed to have died of the plague.

### Amusements and Relaxation.

### NEW PUZZLE PRIZE SCHEME.

**COMMENCED OCTOBER 6th, ENDS DECEMBER 29th, 1888.**

On October the 6th a New Prize Scheme was commenced in these columns.

Prizes will be awarded for the best Original Puzzles in Prose or Verse, relating to any of the Social, Philanthropic, or Political topics of the day, and taking the form of Double Acrostics, Anagrams, Square Words, Decapitations, Transpositions, or any other Puzzles which the ingenuity of the competitors may suggest. (*Answers must accompany contributions.*)

*Readers* are requested to send in weekly the Number of Marks they consider each composition to be entitled to, three being the highest score for any one contribution.

Eight Prizes of Standard Books will be given, the choice of the book to be left to the winner.

First, Second, Third, Fourth, and Fifth Prizes for best Original Puzzles, of £1 1s., 15s., 10s. 6d., 7s. 6d., and 5s. each.

First, Second, and Third Prize for Solutions of 15s., 10s. 6d., and 5s. each.

N.B.—All contributions must reach the office, 38, Old Jewry, E.C., not later than the *First Post* on *Thursday* in each week.

### PUZZLES FOR SOLUTION.

**Fifth Week.**

No. 13. Double Acrostic.

My primals plainly show the plan
My finals should pursue;
A training good for every man,
And every woman too.

Too eagerly sought for, hoarded with care,
Or recklessly squandered—my end is despair.

I sparkle and shine with a brilliancy rare,
Enhancing the beauty of womanhood fair.

I harboured those in ancient days
Who wronged the poor in many ways.

When Nature's laws are thrust aside,
You'll find me scattered far and wide.

Clustered together, and closely combined,
Oft my position remains undefined.

Would you gain me? Labour hard,
I shall then be your reward.

To deal out justice is my care,
And honestly give each his share.

Though not attuned to lofty strains,
My note harmonious sound retains.

Where plants are few and far between,
My hardy creeping growth is seen.

Patience.

No. 14. Original Anagram.

"Nor laugh, in this prim resort."

Tinie.

No. 15. Original Charade.

My first is useful to convey,
To motive power new energy.
My second ladies never tell,
Although they know it very well.
My whole in hospitals is found,
The nurses often take me round.

G. D.

**Marks.**

| Names. | No. of Puzzles sent in Nov. 8. | No. of Marks Nov. 8 | Total of Marks. | Names. | No. of Puzzles sent in Nov. 8. | No. of Marks Nov. 8. | Total of Marks. |
|---|---|---|---|---|---|---|---|
| Patience .... | 3 | 6 | 28 | Hercules .. | 3 | 2 | 11 |
| Lady Clara.. | 3 | 7 | 31 | Gretta ...... | 2 | 4 | 16 |
| Tinie ...... | 3 | 6 | 23 | Dandy Dick | — | — | 5 |
| Bijou ...... | 1 | 3 | 11 | Boadicea .. | — | — | 7 |
| G. D. ...... | 3 | 5 | 11 | Nipper .... | 2 | 6 | 18 |
| Thanet .... | 1 | 2 | 18 | Morico .... | 3 | 7 | 11 |
| Daisy .... | 1 | 3 | 11 | A. M. E..... | — | — | 3 |
| Salford .... | — | — | 6 | John Gilpin | 2 | 4 | 4 |

**Answers to Third Week Puzzles.**

No. 7. Double Acrostic.

K is S
N a P
I ndig O
F O Frog, he would a-wooing go.
E xcursio N

No. 8. Original Anagram.—William Ewart Gladstone.

No. 9. Triple Acrostic.

P eri A sti C

R o N d O

O l D e N

**Marks for Solution of Puzzles.**

Total Marks.—Ruffles (1), 4; Daisy (1), 4; Lady Clara (2), 6; Reldas (1), 5; Mater (1), 3; Condorcet (1), 4; Tinie (2), 5; Gentian, 5; Marguerite, 5; Patience, 2; Gretna (1), 3; Amicus (1), 2; Scrooge (1), 5; Smut, 2; Thanet (1), 7; Jenny Wren (1), 1.

*The figures in parentheses are Marks for the week ending Nov.* 8.

**Conditions.**

I. Every paper sent in must be clearly written, and one side only must be used. All competitors must mark at the head of their papers the number of their competition.

II. Each paper must be signed by the author with his or her real name and address. A *nom de plume* may be added if the writer does not desire to be referred to by us by his real name. In the case of all prizewinners, however, the real name and address will be published.

III. All communications relating to prize competitions should be addressed to "The Prize Editor, The Hospital, 38, Old Jewry, London, E.C." Competitors are requested not to include in letters, etc., to the Prize Editor communications on matters of other business. All letters must be fully prepaid.

IV. The Prize Editor of The Hospital is the sole judge of the competitions, and his decision is in all cases final and without appeal.

V. The Prize Editor reserves the right to publish any paper sent in, whether it receives a prize or not. He does not hold himself responsible for any MSS. sent, nor can he undertake to return rejected competitions.

